# Empathy as a Conditional Resource: System‐Level Moral Distress as an Indirect Pathway in the Associations of Empathy with Burnout and Turnover Intention

**DOI:** 10.1155/jonm/9665722

**Published:** 2026-10-21

**Authors:** Eisho Yoshikawa, Nozomi Sukigara, Yuko Kato, Yuzo Fukuyama, Maya Fukugawa, Michihiko Koeda, Hitomi Kataoka, Amane Tateno

**Affiliations:** ^1^ Department of Medical Psychology Nippon Medical School 1-7-1 Kyonan-cho, Musashino Tokyo 180-0023 Japan nms.ac.jp; ^2^ Department of Neuropsychiatry Nippon Medical School 1-1-5 Sendagi, Bunkyo Tokyo 113-8602 Japan nms.ac.jp; ^3^ Students Counseling Room Nippon Medical School 1-1-5 Sendagi, Bunkyo Tokyo 113-8602 Japan nms.ac.jp; ^4^ Department of Nursing Nippon Medical School Tama Nagayama Hospital 1-7-1 Nagayama, Tama City Tokyo 206-8512 Japan; ^5^ Department of Neuropsychiatry Nippon Medical School Tama Nagayama Hospital 1-7-1 Nagayama, Tama City Tokyo 206-8512 Japan; ^6^ Center for Medical Education and Internationalization Graduate School of Medicine and Faculty of Medicine Kyoto University Yoshida Konoe-cho, Sakyo-ku Kyoto 606-8501 Japan kyoto-u.ac.jp

**Keywords:** burnout, empathy, moral distress, nursing management, turnover intention

## Abstract

**Background:**

Evidence regarding the relationship between empathy and burnout among nurses remains inconsistent. Moral distress may help explain this complexity. Given the associations of moral distress and burnout with turnover intention, it remains insufficiently understood how empathy is linked to these outcomes through multidimensional moral distress, which is a critical concern for nursing management.

**Aim:**

This study examined how empathy relates to burnout and turnover intention among nurses, focusing on indirect associations involving multidimensional moral distress and burnout.

**Methods:**

A cross‐sectional web‐based survey was conducted among Japanese nurses (*n* = 412). Empathy was assessed using the Jefferson Scale of Empathy, burnout using the Burnout Assessment Tool, and moral distress using the Measure of Moral Distress for Healthcare Professionals, which captures multiple dimensions of moral distress. Turnover intention was measured using a single‐item scale. Mediation and moderation analyses were performed.

**Results:**

In the primary parallel mediation analysis, with all four moral distress domains considered simultaneously, positive indirect associations involving system‐level moral distress were statistically significant for burnout and turnover intention using 95% bootstrap confidence intervals. Supplementary separate‐domain sensitivity analyses also identified indirect associations involving clinical‐/patient‐level moral distress. The interaction between empathy and burnout in relation to turnover intention was significant. The negative indirect association of empathy with turnover intention through burnout and the positive serial indirect association through system‐level moral distress and burnout were both conditional on empathy level.

**Conclusions:**

The opposing indirect associations suggest that empathy should not be viewed as a uniformly protective resource in relation to turnover intention. System‐level moral distress showed the most consistent domain‐specific pattern across the analyses. The cross‐sectional design does not establish temporal or causal relationships.

**Implications for Nursing Management:**

Nursing managers may consider staffing adequacy, ethical climate, decision‐making autonomy, and organizational support alongside individual‐focused approaches to empathy and resilience.

## 1. Introduction

Global nursing shortages, recognized as a persistent and systemic barrier to quality care, have been linked to higher levels of burnout, increased turnover intention, and workforce attrition [1, 2]. Rising patient acuity and ongoing resource constraints have further contributed to an exceptionally high‐pressure environment for healthcare professionals. In these ethically and emotionally demanding settings, nurses are expected to make rapid, high‐stakes decisions while managing overwhelming workloads [3, 4]. Nevertheless, the professional standard of providing compassionate, patient‐centered care remains uncompromised, even under extraordinary strain [5, 6].

Empathy is indispensable for delivering high‐quality health care and remains a widely recognized cornerstone of professionalism in medicine [7]. Defined primarily as a cognitive attribute centered on understanding the patient’s perspective and communicating that understanding, clinical empathy plays a vital role in improving trust, adherence, and patient outcomes [5, 8]. Emotional insight, such as recognizing and responding to patients’ emotional states, remains an integral component of empathic care. Thus, empathy in health care entails a balance between intellectual understanding and emotional attunement. This empathic ability is a skill that can be modified through educational intervention, and multiple studies have supported its effectiveness [9, 10].

While empathy is crucial for patient care, its relationship with mental health is complex. In nursing practice, empathy supports psychological well‐being, strengthens the therapeutic relationship, and promotes holistic recovery while enhancing professional satisfaction and care quality [5].

Furthermore, a recent systematic review reported that while empathy is generally negatively associated with burnout, the strength and even direction of this relationship may vary across different subcomponents of both constructs [11]. Despite these findings, the link between empathy and burnout remains insufficiently understood, particularly in terms of the underlying mechanisms. Several mediators, such as guilt [11] and organizational climate [12], have been proposed to mediate the relationship between empathy and burnout. However, a largely overlooked but critical mechanism in this dynamic is moral distress (MD), which arises when empathic nurses, constrained by the organizational environment, are unable to provide high‐quality care in accordance with their moral and professional values [13, 14].

MD is defined as psychological disequilibrium or emotional suffering that arises when clinicians know the ethically appropriate action to take but cannot execute it because of, for example, institutional barriers, hierarchical structures, or limited resources [15, 16]. In contemporary literature, MD is not a transient stressor but a multidimensional phenomenon involving ethical tensions at the individual (e.g., value conflict), interpersonal (e.g., team disagreement), and systemic/organizational (e.g., policy constraints, staffing) levels [14, 15].

The consequences of MD extend far beyond immediate emotional distress. Empirical studies have consistently shown that MD is associated with burnout and other adverse mental health outcomes [6, 17]. Moreover, longitudinal evidence suggests that unresolved MD accumulates over time, increasing the risk for sustained psychological burden [18–20]. Nurses who frequently experience high MD also report greater intention to leave their jobs and lower perceptions of patient safety culture, especially in high‐intensity clinical settings [3, 21].

Although a few studies have examined the association between empathy and MD using validated scales, their findings have been inconsistent [6, 22, 23]. For instance, no significant association was found between empathy and MD among Italian nurses before the COVID‐19 pandemic [23] or among Greek nurses during the pandemic [22]. In contrast, a significant association was reported among Swedish medical care providers, including nurses, during the COVID‐19 pandemic [6]. These studies tend to conceptualize MD as a single, uniform construct, which fails to capture its multifaceted nature. To the best of our knowledge, no such comprehensive study currently exists.

Although associations of empathy with burnout and of MD with burnout and turnover intention have been reported [11, 19, 22], it remains unclear how empathy relates to distinct dimensions of MD and whether these dimensions are involved in the associations of empathy with burnout and turnover intention. In particular, evidence remains limited regarding whether the four domains of the Measure of Moral Distress for Healthcare Professionals (MMD‐HP) show distinct indirect associations when considered concurrently. This study therefore examined associations among empathy, the four dimensions of MD, burnout, and turnover intention using a parallel mediation framework.

## 2. Materials and Methods

A web‐based cross‐sectional survey was conducted in July 2025 in accordance with the Declaration of Helsinki, following approval from the Institutional Central Review Board of Nippon Medical School Foundation (Approval No. M‐2025‐260; May 29, 2025). Electronic informed consent was obtained from all participants. All questionnaires and survey tools used in this study were either publicly available or used with appropriate permission from the original authors or copyright holders.

The target sample size was determined a priori for a multiple linear regression model. Assuming a statistical power of 0.80, an *α* level of 0.05, an anticipated multiple correlation of *R* = 0.20 (*R* ^2^ = 0.04, equivalent to *f* ^2^ = 0.042), and eight predictors, the required sample size was 369. To allow for unusable responses and exclusions during data‐quality screening, the target sample size was set at 400. This sample size calculation was based on the overall regression model. Because the study additionally examined an interaction term and conditional indirect associations, which are not fully represented by a single conventional power calculation, these analyses were treated as exploratory and hypothesis‐generating.

An online research panel served as the sampling frame. Data collection was outsourced to Macromill Inc. (Tokyo, Japan), which operated a nationwide online panel network comprising approximately 1.3 million registered members at the time of the survey. Macromill’s online panels have been used in previous peer‐reviewed studies in Japan, including public health and mental health research employing online panel recruitment procedures [24, 25]. The company manages its research panel and survey data in accordance with marketing‐research standards, including the ICC/ESOMAR International Code.

Eligibility criteria were as follows: (1) age 20–65 years; (2) ability to understand the study information and complete the questionnaire in Japanese; (3) current full‐time employment as a nurse providing nursing care in a hospital with at least 20 beds in Japan; and (4) provision of electronic informed consent. Exclusion criteria for survey responses were based on Macromill’s established quality‐control procedures for identifying responses considered likely to be invalid or of insufficient quality. These criteria included the following: (1) duplicate participation by the same panel member, which was prevented by the online‐survey system; (2) straight‐lining, defined as selecting the same response option across 12 or more consecutive items in a matrix‐format question; and (3) an unusually short questionnaire completion time, as determined using the provider’s proprietary short‐completion‐time detection algorithm (Japanese Patent No. 4,795,496).

The questionnaire was administered using Macromill’s AIRs online‐survey system. Respondents were required to answer all displayed mandatory items before the questionnaire could be classified as complete.

From its nationwide online panel network, the survey provider randomly selected approximately 30,000 members aged 20–65 years through a computerized procedure. Before distributing invitations, the provider confirmed that the invitation pool did not show marked imbalance with respect to geographic region, age group, or sex. Although panel members were selected at random within the panel, the panel itself comprises individuals who had voluntarily registered with the provider; the resulting sample is therefore a nonprobability sample. No stratification or quota sampling was applied at either the invitation or the enrollment stage.

To reduce the possibility that the sample would be disproportionately composed of early responders with particular occupational or lifestyle characteristics, the number and timing of survey invitations were adjusted by the provider’s system throughout the field period. Invitations were distributed through email or the provider’s survey application, and respondents could participate using either a computer or a smartphone. Potential participants first completed an online screening survey. Respondents who met the eligibility criteria were automatically directed to the study information and consent pages and, after providing electronic informed consent, to the main questionnaire. Respondents who did not meet the eligibility criteria were automatically directed to the survey‐termination page. Participants received Macromill points as compensation according to the survey company’s standard incentive scheme (1 point = JPY 1): up to 2 points for completing the screening survey and up to 60 points for completing the main questionnaire. Eligible respondents were enrolled in the order in which they completed the questionnaire. Duplicate participation by the same panel member was prevented by the survey system. The contracted target sample size was 400 participants; however, the provider collected responses beyond this target to allow for exclusions during data‐quality screening. After the initial quality‐control procedures, 424 eligible and complete responses remained. Questionnaire completion time was then assessed using the provider’s proprietary short‐completion‐time detection algorithm (Japanese Patent No. 4,795,496), and 12 unusually rapid responses were excluded. Consequently, 412 valid, de‐identified responses were transferred to the research team. All 412 responses were included in the primary analyses, and no additional responses were excluded by the research team.

### 2.1. Demographic and Occupational Characteristics

The questionnaire survey collected demographic information (age, sex, marital status, having children or not), occupational characteristics (current job title/position, department/unit type [inpatient ward vs. outpatient clinic], total years of nursing experience, shift work status, and the number of hospital beds), and lifestyle information (smoking and alcohol consumption).

### 2.2. Measures

Empathy was assessed using the Japanese version of the Jefferson Scale of Empathy–Health Professionals Version (JSE) [26], developed based on the original English version [7]. The JSE conceptualizes empathy primarily as a relatively stable professional orientation toward patient care rather than a transient emotional state. The 20‐item instrument is rated on a 7‐point Likert scale (1 = *strongly disagree*, 7 = *strongly agree*). Total scores range from 20 to 140, with higher scores indicating a more empathic orientation toward patient care. In the initial Japanese validation of the student version, factor analysis reproduced the three expected components, all item–total correlations were positive and statistically significant, and Cronbach’s *α* was 0.80 [26]. More recently, a nationwide Japanese study including 5441 physicians and 965 nurses reported good internal consistency for the JSE in the combined sample (Cronbach’s *α* = 0.89) [27].

MD was assessed using the Japanese version of the Measure of Moral Distress for Healthcare Professionals (MMD‐HP) [28], translated and validated from the original English version [15]. The 27‐item MMD‐HP describes situations that might cause MD. Each item is rated by two 5‐point Likert scales: frequency (0 = *never*, 4 = *very frequently*) and distress level (0 = *none*, 4 = *great extent*). Each item’s composite score is calculated by multiplying the frequency and intensity ratings. The total score ranges from 0 to 432, with higher scores indicating greater MD. The MMD‐HP consists of four factors: system‐level, clinical‐/patient‐level, team/personal threat or vulnerability level, and team/patient interaction level. The Japanese validation study reported Cronbach’s *α* = 0.91 (95% confidence interval [CI], 0.89–0.92); the four‐factor confirmatory model yielded a chi‐square‐to‐degrees‐of‐freedom ratio (CMIN/df) of 2.14 and a root mean square error of approximation (RMSEA) of 0.061 (90% CI, 0.055–0.067), and hypothesized associations with clinical experience and intention to leave supported construct validity [28].

Burnout was measured using the Japanese version of the Burnout Assessment Tool (BAT) [29]. The 23‐item BAT assesses four core dimensions: exhaustion, mental distance, emotional impairment, and cognitive impairment. Items are rated on a 5‐point Likert scale (1 = *never*, 5 = *always*), with higher scores indicating higher levels of burnout. Total scores range from 23 to 115, with higher scores reflecting greater burnout severity. For the 23‐item core Japanese BAT used here, the validation study reported Cronbach’s *α* = 0.96 and 1‐month test–retest reliability of *r* = 0.64; a correlated four‐factor model yielded a comparative fit index (CFI) of 0.93, a Tucker–Lewis index (TLI) of 0.92, and RMSEA = 0.06 [29].

Turnover intention was assessed using a single item developed for this study: “I have thought about leaving my current job.” Participants rated the extent to which they agreed or disagreed with this statement on a 7‐point Likert scale (1 = *strongly disagree*, 7 = *strongly agree*), with higher scores indicating stronger agreement. Each participant selected only one response option by marking the corresponding circle.

Although most of these instruments do not uniformly specify explicit time frames, they differ conceptually in what they assess: Empathy reflects a professional disposition, MD captures experiences of ethical distress embedded in clinical practice, and burnout represents a work‐related psychological state. This conceptual distinction informed the ordering of variables in the hypothesized analytical model but does not establish temporal precedence.

### 2.3. Statistical Analyses

Analyses were conducted using IBM SPSS Statistics (Version 27; IBM Corp., Armonk, NY), supplemented by R‐based analyses as described below. A two‐tailed alpha level of 0.05 was used to determine statistical significance. Descriptive statistics were first computed for all study variables, and internal consistency was evaluated for all multi‐item instruments using Cronbach’s *α*. Bivariate analyses were conducted to examine associations among key variables. For continuous variables, Pearson’s or Spearman’s correlations were used as appropriate based on distributional properties. Because turnover intention was assessed with a single seven‐point item and is therefore ordinal, Spearman’s rank‐order correlations were used for all associations involving this variable. Because the item captured graded turnover intention across seven well‐represented response categories, it was treated as approximately continuous in the primary regression and conditional process analyses; a cumulative proportional‐odds ordinal logistic regression was used as a sensitivity analysis. In that analysis, each continuous predictor column, including the centered BAT × JSE product, was separately standardized to unit standard deviation; *p* values were obtained from likelihood‐ratio tests.

We employed the PROCESS macro (Version 5.0) for SPSS [30] to test our hypothesized mediation models. To assess the potential for common method bias, Harman’s single‐factor test was conducted. All measured items were subjected to an exploratory factor analysis using an unrotated solution.

### 2.4. Parallel Mediation Framework as the Primary Analysis

First, we conducted a mediation analysis using PROCESS Model 80 (Figure 1) [30] to examine the mediating roles of the four MMD‐HP subscales in relation to JSE, BAT, and turnover intention. In this model, turnover intention was specified as the dependent variable, empathy as the independent variable, the four MMD‐HP subscales as parallel mediators, and burnout as a sequential mediator.

**FIGURE 1:**
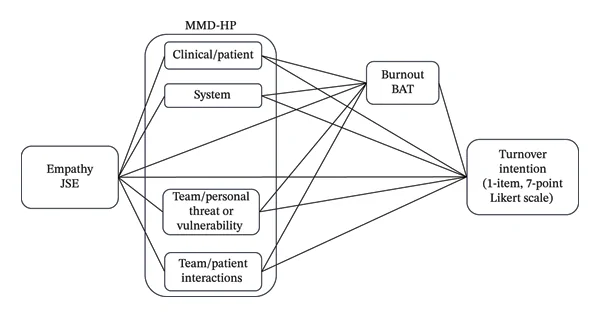
Conceptual model illustrating the associations among empathy, multiple dimensions of moral distress, burnout, and turnover intention. BAT: Burnout Assessment Tool; JSE: Jefferson Scale of Empathy; MMD‐HP: Measure of Moral Distress for Healthcare Professionals. Turnover intention was assessed using a single item on a 7‐point Likert scale.

This mediation analysis consisted of three sets of regression equations. The first regression assessed the association between empathy and each subscale of MD. In these analyses, each MMD‐HP subscale was entered as the dependent variable and JSE as the independent variable. The second regression examined the association between empathy and burnout. BAT was entered as the dependent variable, JSE as the independent variable, and MMD‐HP subscales as covariates. The third regression assessed the association between burnout and turnover intention. Turnover intention was entered as the dependent variable, burnout as the independent variable, and MMD‐HP subscales and JSE as covariates. The four MMD‐HP domains were entered simultaneously in the downstream models to estimate the domain‐specific association of each dimension after accounting for variance shared with the other domains. Because the MMD‐HP domains were correlated, variance inflation factors (VIFs) were examined to assess multicollinearity.

Second, to test the assumption of homogeneity of regression slopes, we exploratorily examined potential interaction effects involving empathy. As a significant BAT × JSE interaction was detected (see Supporting Tables S1 and S2), we proceeded to the final step by estimating conditional indirect associations using PROCESS Model 4, with the X‐by‐M interaction option applied as appropriate. Significant interactions were visualized using simple slopes analysis and the Johnson–Neyman technique to identify regions of significance, implemented using PROCESS Model 1. For the indirect association analyses, each MMD‐HP subdomain was first regressed on JSE. BAT was subsequently regressed on JSE and all four MMD‐HP subdomains, whereas turnover intention was regressed on JSE, all four MMD‐HP subdomains, BAT, and the BAT × JSE interaction. An R‐based analysis was used to estimate the conditional serial indirect associations through each MMD‐HP subdomain and BAT within this parallel mediation framework. Conditional associations were evaluated at JSE values of 79, 86, and 108, corresponding to the 16th, 50th, and 84th percentiles in the complete‐case sample (*n* = 367); these probe values were held fixed across analyses. The 95% percentile bootstrap CIs were obtained from 5000 nonparametric case‐resampling bootstrap samples.

Additional sensitivity analyses were conducted using hierarchical regression models with HC3 heteroskedasticity‐consistent standard errors, in which turnover intention was entered as the dependent variable. Model 1 included empathy, the four MD subdomains, burnout, and the burnout × empathy interaction adjusted for age and sex; Model 2 further adjusted for marital and parental status; and Model 3 additionally included hospital size, night‐shift work, and inpatient setting.

Nursing experience and managerial position were structurally missing for 45 participants because of a screening‐system routing discrepancy. Models 1–3 were therefore estimated in the full sample of 412 participants, whereas a final complete‐case model (Model 4), additionally including managerial position and nursing experience of 15 years or more, was estimated among the 367 participants with complete data on both variables. Age was retained in Model 4, and all VIFs were below 5. For the indirect association sensitivity analyses, the model specification corresponding to each main indirect association analysis was retained, and the same cumulative demographic and occupational covariate sets were added. Full results are presented in Supporting Tables S3 and S4.

### 2.5. Separate‐Domain Sensitivity Analyses

As a further sensitivity analysis, each MMD‐HP domain was examined separately, without the other MMD‐HP domains, using the corresponding analytic specification. Separate turnover intention regression models included JSE, BAT, and the BAT × JSE interaction and were estimated using HC3 heteroskedasticity‐consistent standard errors. To conservatively account for the four domain‐specific comparisons, a Bonferroni correction (*α* = 0.05/4 = 0.0125) was applied; adjusted *p* values were calculated from unrounded *p* values. For indirect and conditional serial indirect associations, both conventional 95% and Bonferroni‐adjusted 98.75% bootstrap CIs were examined.

ChatGPT (OpenAI, San Francisco, CA, USA) was used for language editing, improvement of readability, and computational consistency checks. After using this tool, the authors reviewed and edited the content as needed and take full responsibility for the content of the publication.

## 3. Results

Harman’s single‐factor test indicated that the first unrotated factor accounted for 20.04% of the total variance.

### 3.1. Participant Characteristics

Table 1 presents the demographic and occupational characteristics of the participants. Mean age was 40.2 years (standard deviation [SD] = 11.6). Mean (SD) scores were 90.41 (14.60) for empathy (JSE; *α* = 0.841), 112.37 (68.14) for MD (MMD‐HP total; *α* = 0.951), 67.51 (15.10) for burnout (BAT; *α* = 0.921), and 4.28 (1.81) for turnover intention (single item). Cronbach’s *α* for the four MMD‐HP subdomains ranged from 0.782 to 0.880. Descriptive statistics and internal consistency coefficients are presented in Table 2.

**TABLE 1:** Demographic characteristics of participants.

Variable	Category	Mean	(SD)
Age (years), mean (SD)		40.2	(11.6)

		***n***	**(%)**

Age (years)	< 25	20	(5)
	25–34	144	(35)
	35–44	95	(23)
	≥ 45	153	(37)

Gender	Male	190	(46)
	Female	222	(54)

Unit type	Outpatient clinic	132	(32)
	Inpatient ward	214	(52)
	Other	66	(16)

Working position	Head nurse	56	(14)
	General nurse	311	(75)
	Missing	45	(11)

Years of nursing work	≤ 4	43	(10)
	5–8	94	(23)
	9–14	68	(17)
	≥ 15	162	(39)
	Missing	45	(11)

Night‐shift work	No	135	(33)
	Yes	277	(67)

Number of hospital beds	20‐99	125	(30)
	100‐499	216	(52)
	≥ 500	71	(17)

Marital status	Unmarried	176	(43)
	Married	236	(57)

Children	No	193	(47)
	Yes	219	(53)

Smoking	No	335	(81)
	Yes	77	(19)

Alcohol	No	166	(40)
	Yes	246	(60)

**TABLE 2:** Descriptive statistics and internal consistency of study variables (*N* = 412).

Variable	*M*	SD	*α*
Empathy (JSE)	90.41	14.60	0.841
Moral distress (MMD‐HP, total product score)	112.37	68.14	0.951
Clinical‐/patient‐level MD	29.02	18.48	0.839
System‐level MD	36.53	23.97	0.880
Team/personal threat (TPV‐MD)	26.74	19.03	0.865
Team/patient interaction (TPI‐MD)	20.08	13.20	0.782
Burnout (BAT)	67.51	15.10	0.921
Turnover intention^a^	4.28	1.81	—

*Note: α* = Cronbach’s alpha. MMD‐HP subdomain scores are product scores (frequency × intensity); subdomain reliability coefficients are reported descriptively, as the MMD‐HP was primarily designed to yield a total moral distress score.

Abbreviations: BAT = Burnout Assessment Tool, JSE = Jefferson Scale of Empathy, MD = moral distress, MMD‐HP = Measure of Moral Distress for Healthcare Professionals.

^a^Single item rated from 1 (*strongly disagree*) to 7 (*strongly agree*); internal consistency coefficients are not applicable.

### 3.2. Bivariate Associations

Table 3 presents results of bivariate associations. There were significant positive associations between JSE and clinical‐level and system‐level MMD‐HP and a significant negative association with burnout. Turnover intention was positively correlated with MMD‐HP across all subscales and the total score as well as with BAT but was not significantly correlated with JSE. MMD‐HP subscales were significantly intercorrelated.

**TABLE 3:** Bivariate correlations among empathy (JSE), multidimensional moral distress (MMD‐HP), burnout (BAT), and turnover intention (TI).

	JSE	MMD‐HP					BAT
		Clin‐MD	Sys‐MD	TPV‐MD	TPI‐MD	MMD‐HP total score	
Clin‐MD	0.153^∗∗^						
Sys‐MD	0.179^∗∗^	0.816^∗∗^					
TPV‐MD	−0.030	0.728^∗∗^	0.751^∗∗^				
TPI‐MD	0.022	0.770^∗∗^	0.774^∗∗^	0.806^∗∗^			
MMD‐HP total score	0.100^∗^	0.911^∗∗^	0.933^∗∗^	0.897^∗∗^	0.900^∗∗^		
BAT	−0.213^∗∗^	0.300^∗∗^	0.367^∗∗^	0.391^∗∗^	0.310^∗∗^	0.380^∗∗^	
TI	0.050	0.274^∗∗^	0.326^∗∗^	0.216^∗∗^	0.216^∗∗^	0.307^∗∗^	0.353^∗∗^

*Note:* JSE: Jefferson Scale of Empathy–Health Professions Version; Clin‐MD: clinical/patient‐level moral distress; Sys‐MD: system‐level moral distress; TPV‐MD: team/personal threat or vulnerability moral distress; TI: turnover intention; Pearson’s correlations are reported except for associations involving TI, which use Spearman’s rank correlations.

Abbreviations: BAT, Burnout Assessment Tool; MMD‐HP, Measure of Moral Distress for Healthcare Professionals; TPI‐MD, team/patient interaction moral distress.

^∗∗^ *p* < 0.01 (two‐tailed).

^∗^ *p* < 0.05 (two‐tailed).

### 3.3. Mediation Analysis

In the first regression analysis (Table 4a), JSE was significantly and positively associated with system‐ and clinical‐level MD in MMD‐HP, whereas associations with other MD domains were nonsignificant. In the second regression analysis (Table 4b), JSE showed a significant negative association with BAT, whereas system‐level MMD‐HP and team/personal threat or vulnerability were significantly and positively associated with BAT. In the third regression analysis (Table 4c), turnover intention showed no significant association with JSE, whereas BAT and system‐level MMD‐HP were significantly associated with turnover intention. VIFs for the predictors in the primary BAT and turnover intention models ranged from 1.12 to 4.14 (Table 4), with all values below 5.

**TABLE 4:** Regression coefficients for the conditional process model examining the associations among empathy (JSE), multidimensional moral distress, burnout, and turnover intention.

	Independent variables	*R* ^2^	*B*	SE	*β*	*t*	*p*	LLCI	ULCI	VIF
*(a) Dependent Variable: Mediator*										
Clin‐MD	JSE	0.023	0.19	0.062	0.15	3.14	0.002	0.072	0.32	—
Sys‐MD	JSE	0.032	0.29	0.08	0.18	3.68	< 0.001	0.14	0.45	—
TPV‐MD	JSE	0.0009	−0.039	0.064	−0.03	−0.6	0.549	−0.17	0.088	—
TPI‐MD	JSE	0.0005	0.02	0.045	0.02	0.45	0.654	−0.068	0.11	—

*(b) Dependent Variable: Secondary mediator*		*0.232*								
BAT	JSE		−0.273	0.048	−0.26	−5.72	< 0.001	−0.367	−0.179	1.12
	Clin‐MD		−0.016	0.067	−0.02	−0.23	0.817	−0.148	0.117	3.60
	Sys‐MD		0.224	0.054	0.36	4.13	< 0.001	0.117	0.331	3.92
	TPV‐MD		0.193	0.064	0.24	3.03	0.003	0.068	0.318	3.41
	TPI‐MD		−0.161	0.097	−0.14	−1.65	0.099	−0.351	0.03	3.81

*(c) Dependent Variable: Final outcome*		*0.157*								
TI	JSE		−0.002	0.006	−0.01	−0.26	0.794	−0.014	0.011	1.21
	Clin‐MD		0.005	0.008	0.06	0.64	0.523	−0.011	0.022	3.60
	Sys‐MD		0.022	0.007	0.29	3.12	0.002	0.008	0.035	4.09
	TPV‐MD		−0.009	0.008	−0.09	−1.08	0.282	−0.025	0.007	3.49
	TPI‐MD		−0.011	0.012	−0.08	−0.87	0.387	−0.035	0.013	3.83
	BAT		0.032	0.006	0.27	5.2	< 0.001	0.02	0.045	1.30

*(d) With interaction item*		*0.175*								
TI	JSE		0.003	0.007	0.02	0.41	0.682	−0.011	0.017	1.29
	Clin‐MD		0.005	0.009	0.05	0.55	0.579	−0.012	0.022	3.60
	Sys‐MD		0.019	0.008	0.26	2.50	0.013	0.004	0.034	4.14
	TPV‐MD		−0.012	0.008	−0.12	−1.40	0.163	−0.028	0.005	3.54
	TPI‐MD		−0.005	0.012	−0.04	−0.43	0.665	−0.029	0.018	3.92
	BAT		0.033	0.007	0.28	4.87	< 0.001	0.020	0.047	1.31
	BATc × JSEc		0.0011	0.0004	0.14	2.67	0.008	0.0003	0.0019	1.12

*Note:* BATc: mean‐centered BAT; JSE: Jefferson Scale of Empathy–Health Professions Version; JSEc: mean‐centered JSE; Clin‐MD: clinical/patient‐level moral distress; Sys‐MD: system‐level moral distress; TPV‐MD: team/personal threat or vulnerability moral distress; TI: turnover intention; B: unstandardized regression coefficient; β: standardized coefficient; LLCI: lower limit of the 95% confidence interval; ULCI: upper limit of the 95% confidence interval.

Abbreviations: BAT, Burnout Assessment Tool; MMD‐HP, Measure of Moral Distress for Healthcare Professionals; SE, standard error; TPI‐MD, team/patient interaction moral distress.

Table 4d shows the results of regression analyses examining BAT, JSE, and their interactions (BATc × JSEc) for turnover intention. Turnover intention was positively associated with BAT, system‐level MMD‐HP, and the BAT × JSE interaction. Heteroskedasticity was detected based on the Breusch–Pagan test, and parameter estimates were calculated using heteroskedasticity‐consistent standard errors (HC3). Simple slope analyses indicated that the positive association between BAT and turnover intention was stronger at higher levels of JSE (Figure 2). The Johnson–Neyman analysis further showed that the conditional association between BAT and turnover intention became statistically significant when JSE scores exceeded 76.66 (Figure 3). This boundary was approximately 0.94 standard deviations below the sample mean. In the observed sample, 370 of the 412 participants (89.8%) had JSE scores above this boundary. The ordinal logistic sensitivity analysis corresponding to the primary simultaneous specification reproduced the focal pattern of the linear model: System‐level MD (odds ratio [OR] = 1.87, *p* < 0.001), burnout (OR = 1.76, *p* < 0.001), and the BAT × JSE interaction (OR = 1.37, *p* = 0.0017) remained statistically significant, whereas the other MMD‐HP domains did not.

**FIGURE 2:**
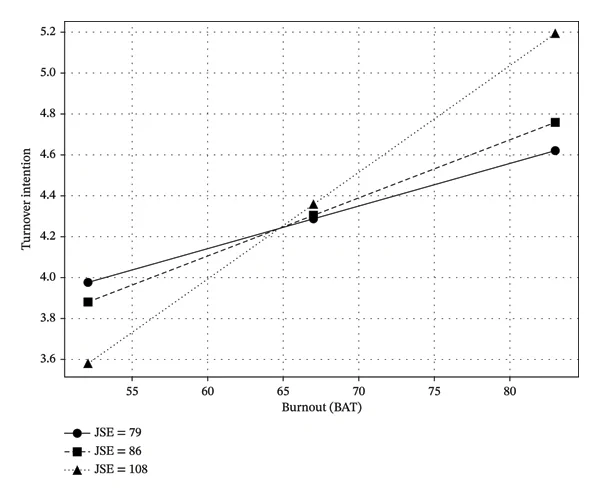
Simple slopes showing the moderating role of empathy (JSE) in the association between burnout (BAT) and turnover intention. BAT: Burnout Assessment Tool; JSE: Jefferson Scale of Empathy. Simple slopes were estimated at JSE values of 79, 86, and 108, corresponding to the 16th, 50th, and 84th percentiles in the complete‐case sample (*n* = 367); these probe values were held fixed across analyses.

**FIGURE 3:**
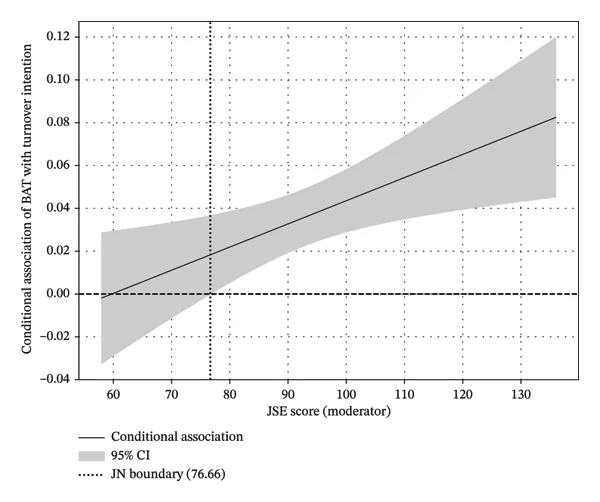
Johnson–Neyman plot showing the conditional association between burnout (BAT) and turnover intention across levels of empathy (JSE). The solid line represents the estimated conditional association, and the shaded region represents its 95% confidence interval. The vertical dashed line indicates the Johnson–Neyman significance boundary (JSE = 76.66), above which the positive conditional association between burnout and turnover intention becomes statistically significant. Conditional associations were estimated using PROCESS Model 1 with HC3 heteroskedasticity‐consistent standard errors. BAT = Burnout Assessment Tool; JSE = Jefferson Scale of Empathy.

### 3.4. Indirect Associations

#### 3.4.1. Parallel Mediation Framework as the Primary Analysis

Table 5 presents indirect associations between JSE and BAT and those between JSE and turnover intention via the MMD‐HP subscales. While the total indirect association between JSE and BAT was not statistically significant, there was a significant total indirect association between JSE and turnover intention via all MMD‐HP subscales. The bootstrap analysis revealed a significant positive indirect association between JSE and BAT via system‐level MMD‐HP, as well as a significant positive indirect association between JSE and turnover intention via system‐level MMD‐HP.

**TABLE 5:** Indirect associations through mediators.

Indirect association	*B*	Boot SE	Boot LLCI	Boot ULCI	*β*
Total (JSE to all mediators to BAT)	0.0521	0.031	−0.0062	0.1174	0.0504
JSE to BAT via Clin‐MD	−0.003	0.014	−0.032	0.0255	−0.0029
JSE to BAT via Sys‐MD	0.0658	0.0262	0.0217	0.1218	0.0636
JSE to BAT via TPV‐MD	−0.0074	0.0165	−0.0414	0.0266	−0.0072
JSE to BAT via TPI‐MD	−0.0032	0.0098	−0.027	0.0153	−0.0031

Total (JSE to all mediators to TI)	0.0069	0.0026	0.0018	0.0118	0.0561
JSE to TI via Clin‐MD	0.0009	0.0019	−0.0026	0.0051	0.0077
JSE to TI via Sys‐MD	0.0056	0.0029	0.0008	0.0120	0.0456
JSE to TI via TPV‐MD	0.0004	0.0012	−0.0020	0.0030	0.0036
JSE to TI via TPI‐MD	−0.0001	0.0007	−0.0019	0.0012	−0.0008

*Note:* Values represent unstandardized bootstrap estimates based on 5000 resamples. JSE: Jefferson Scale of Empathy–Health Professions Version; Clin‐MD: clinical‐/patient‐level moral distress; Sys‐MD: system‐level moral distress; TPV‐MD: team/personal threat or vulnerability moral distress; TI: turnover intention; B: unstandardized regression coefficient; β: standardized coefficient; LLCI: lower limit of the 95% confidence interval; ULCI: upper limit of the 95% confidence interval.

Abbreviations: BAT, Burnout Assessment Tool; SE, standard error; TPI‐MD, team/patient interaction moral distress.

Table 6 presents the conditional indirect association between JSE and turnover intention through burnout (BAT) and the conditional serial indirect associations through each MMD‐HP domain and burnout, estimated at JSE values of 79, 86, and 108.

**TABLE 6:** Conditional indirect associations between empathy and turnover intention.

	JSE value	*B*	Boot SE	Boot LLCI	Boot ULCI
*Via burnout (JSE ⟶ BAT ⟶ TI)*					
16th percentile	79	−0.006	0.0025	−0.0113	−0.0015
50th percentile	86	−0.008	0.0024	−0.0135	−0.0039
84th percentile	108	−0.0145	0.004	−0.0236	−0.0078

*Via clinical-/patient-level MMD and burnout*					
16th percentile	79	−0.0001	0.0003	−0.0008	0.0006
50th percentile	86	−0.0001	0.0004	−0.0010	0.0008
84th percentile	108	−0.0002	0.0007	−0.0017	0.0014

*Via system-level MMD and burnout*					
16th percentile	79	0.0014	0.0008	0.0002	0.0033
50th percentile	86	0.0019	0.0009	0.0006	0.0039
84th percentile	108	0.0034	0.0014	0.0012	0.0067

*Via team/personal threat MMD and burnout*					
16th percentile	79	−0.0002	0.0004	−0.0010	0.0007
50th percentile	86	−0.0002	0.0005	−0.0013	0.0008
84th percentile	108	−0.0004	0.0009	−0.0024	0.0014

*Via team/patient interaction MMD and burnout*					
16th percentile	79	−0.0001	0.0002	−0.0007	0.0003
50th percentile	86	−0.0001	0.0003	−0.0008	0.0004
84th percentile	108	−0.0002	0.0005	−0.0014	0.0008

*Note:* JSE: Jefferson Scale of Empathy–Health Professions Version; MMD, Measure of Moral Distress for Healthcare Professionals; TI, turnover intention; B: unstandardized regression coefficient; LLCI/ULCI: lower/upper limit of 95% bootstrap confidence intervals (5000 resamples). For the domain‐specific serial pathways, each MMD domain was regressed separately on JSE at the first stage; all four MMD domains were entered in the BAT and TI models, with BAT × JSE retained in the TI model. The conditional serial indirect associations were estimated within the parallel mediation framework. JSE values of 79, 86, and 108 are the 16th, 50th, and 84th percentiles in the complete‐case sample (*n* = 367) and were held fixed across analyses.

Abbreviations: BAT, Burnout Assessment Tool; SE, standard error.

The indirect association between JSE and turnover intention through burnout was statistically significant and negative and increased in absolute magnitude at higher JSE values. Among the domain‐specific serial indirect associations, only the positive association through system‐level MMD‐HP and burnout was statistically significant at all three JSE values; the corresponding serial indirect associations through the other MMD‐HP domains were not statistically significant.

In the covariate‐adjusted sensitivity analyses, system‐level MD and burnout remained significantly associated with turnover intention across Models 1–4, and the burnout × empathy interaction remained significant across all four models (Supporting Table S3). Among the demographic and occupational covariates, only marital status was significantly associated with turnover intention (Models 2 and 3); this association was not observed in Model 4.

The indirect associations were also generally robust to covariate adjustment (Supporting Table S4). The specific indirect association through system‐level MD remained statistically significant across all adjustment specifications. The conditional indirect association through burnout remained statistically significant at JSE = 86 and 108 across all models and at JSE = 79 in Models 1–3; at JSE = 79 in Model 4, the 95% CI included zero (−0.009836 to 0.000551). The serial indirect association through system‐level MD and burnout remained statistically significant at JSE values of 86 and 108 across all models and at JSE = 79 in Models 1–3; at JSE = 79 in Model 4, the 95% CI narrowly included zero. The corresponding serial indirect associations through the other MMD‐HP domains were not statistically significant (Supporting Table S4).

#### 3.4.2. Separate‐Domain Sensitivity Analyses

When each MMD‐HP domain was examined in a separate turnover intention regression model, clinical‐/patient‐level MD (*B* = 0.0124, *p* = 0.0238) and system‐level MD (*B* = 0.0132, *p* = 0.0029) were statistically significant at the conventional level, whereas the two team‐related domains were not. After Bonferroni correction for the four domain‐specific tests (*α* = 0.0125), only the system‐level association remained statistically significant. In the separate‐domain analyses, the JSE ⟶ MMD ⟶ BAT indirect associations through clinical‐/patient‐level and system‐level MD both remained statistically significant using Bonferroni‐adjusted 98.75% bootstrap CIs. For JSE ⟶ MMD ⟶ turnover intention, only the system‐level indirect association remained statistically significant after correction. For the conditional serial associations through MMD and BAT, both clinical‐/patient‐level and system‐level pathways remained statistically significant at JSE = 86 and 108 using the 98.75% intervals, whereas at JSE = 79, the clinical‐/patient‐level interval narrowly excluded zero and the system‐level interval included zero. Neither team‐related domain showed significant indirect or conditional serial indirect associations (Supporting Table S5).

## 4. Discussion

This web‐based, cross‐sectional study examined relationships among empathy, MD, burnout, and turnover intention using a comprehensive mediation framework that incorporated the multidimensional structure of MD. Several statistically meaningful patterns were observed. Empathy was directly and negatively associated with burnout, with no significant direct association with turnover intention. Among the subdimensions of MD, system‐level and clinical‐/patient‐level MD were positively associated with empathy. However, when the four MMD‐HP domains were considered concurrently in the downstream models, system‐level MD showed the most consistent domain‐specific pattern. Empathy showed significant positive indirect associations with turnover intention through system‐level MD, both with and without burnout as a subsequent mediator. Separate‐domain sensitivity analyses additionally identified some indirect associations involving clinical‐/patient‐level MD, indicating that the system‐level finding should not be interpreted as exclusive.

Burnout was positively associated with turnover intention, with this association stronger at higher levels of empathy. The Johnson–Neyman boundary indicated that the positive conditional association between burnout and turnover intention was statistically significant across most of the observed empathy distribution rather than only among nurses with exceptionally high empathy. Conversely, empathy showed a negative indirect association with turnover intention through lower burnout. This negative indirect association increased in absolute magnitude at higher empathy levels, as did the positive serial indirect association through system‐level MD and burnout. Together, these findings suggest that opposing indirect associations and moderation may coexist, highlighting the complexity of the relationship between empathy and turnover intention.

For context, previous studies of practicing nurses in Asian countries have reported mean JSE scores ranging from 87.5 in Iran to approximately 110.6 in Taiwan and Japan [27, 31–33], and the mean score in the present study (90.4) falls within this range. Direct comparisons across studies are limited, however, by differences in sampling frames, participant characteristics, and survey procedures. Importantly, the large proportion of participants above the Johnson–Neyman boundary does not indicate that empathy scores were unusually high in absolute terms; rather, it reflects the relatively low location of the model‐derived boundary within the observed JSE distribution [34].

The findings of the present study help clarify previously inconsistent results regarding the relationship between empathy and MD [6, 22, 23]. Several methodological and contextual factors may explain these divergent findings. Notably, unlike those previous studies, which conceptualized MD as a unidimensional construct, the present study employed a multidimensional framework that allowed the four domains to be considered concurrently. In the simultaneous analyses, system‐level MD showed the most consistent domain‐specific pattern, although separate‐domain sensitivity analyses also identified some associations involving clinical‐/patient‐level MD.

Another possibility is that contextual moderators shape the association between empathy and MD, including differences in healthcare systems and historical timing. For example, prepandemic studies conducted in Italy primarily captured MD arising from discrete clinical dilemmas, whereas pandemic‐era research highlighted a shift toward chronic, system‐driven ethical challenges [6, 22, 23]. Together, these findings suggest that inconsistencies in the literature may reflect contextual variation rather than true contradictions.

Regarding the association between empathy and burnout, previous studies have proposed several mediators through which empathy is positively associated with burnout, including intensive care unit–related stressors, organizational climate, guilt, and secondary traumatic stress [12, 17, 35–37]. The present findings extend this literature by highlighting system‐level MD as a particularly salient dimension in the associations among empathy, burnout, and turnover intention. System‐level MD arises when persistent organizational constraints prevent healthcare professionals from acting in accordance with their ethical judgment and professional values, often in ways that cannot be resolved through individual effort [16, 28]. Unlike MD rooted in discrete clinical encounters, this form of distress is embedded in chronic institutional conditions and tends to accumulate over time, contributing to emotional exhaustion and disengagement [17].

One possible explanation for the positive association between empathy and system‐level MD is that highly empathic nurses may be especially sensitive to such structural contradictions. Importantly, the present findings do not indicate that empathy itself is problematic; rather, the positive association with system‐level MD may reflect greater awareness of organizational constraints that hinder empathic, patient‐centered care. System‐level MD was also directly associated with turnover intention, independent of burnout; one possible interpretation is that consideration of leaving may sometimes be related to perceived value incongruence in organizational environments rather than exhaustion alone. Notably, empathy showed a negative direct association with burnout alongside a positive indirect association through system‐level MD. These opposing associations highlight the importance of distinguishing individual pathways when interpreting the relationship between empathy and burnout. Their coexistence suggests that the overall relationship among empathy, burnout, and turnover intention may vary across individual and contextual conditions.

The positive association between burnout and turnover intention was stronger at higher empathy levels. The negative indirect association through burnout and the positive serial indirect association through system‐level MD and burnout share the same conditional burnout–turnover–intention slope. Thus, the observed pattern of direct, indirect, and conditional associations does not indicate an overall protective or harmful effect of empathy.

These conditional associations may not be uniform across situations. External pressures, such as a pandemic, may be associated with higher system‐level MD, and individual characteristics may also modify the associations among empathy, burnout, and turnover intention. Burnout may additionally arise in relation to factors beyond empathy. In such contexts, the observed association between burnout and turnover intention may differ across levels of empathy. These possibilities should be regarded as tentative, theory‐informed interpretations that require longitudinal investigation.

Taken together, these findings suggest that empathy may be relevant not only to its direct and indirect associations with burnout but also to the conditional association between burnout and turnover intention. The Johnson–Neyman analysis indicated that the positive association between burnout and turnover intention was statistically significant across most of the observed empathy distribution. From a nursing management perspective, one possible interpretation is that, as empathic orientation becomes more deeply embedded in professional values and identity, experiences of exhaustion and depletion may be understood not only as personal strain but also in relation to nurses’ perceived inability to provide the patient‐centered care they value. This interpretation of burnout may help explain the stronger association between burnout and turnover intention at higher empathy levels, as burnout may be experienced not only as emotional depletion but also as a perceived inability to uphold professional values and caregiving standards. Because professional identity, value incongruence, and reasons for considering turnover were not directly assessed, this interpretation should be regarded as tentative rather than as evidence of a clinical threshold or causal mechanism [11].

This interpretation may be understood in light of the role of empathy in nursing, not merely as an interpersonal communication skill, but as a core dimension of professional identity and ethical orientation [38]. Empathy has been discussed as contributing to professional identity formation by shaping how healthcare professionals derive meaning from patient care and understand their professional roles [39]. Nurses with higher levels of empathy may therefore be more likely to internalize professional and ethical values as integral to their caregiving. When they experience burnout, it may be perceived not simply as physical or emotional exhaustion, but as a threat to their professional self‐concept, namely, their perceived ability to deliver the standard of care they believe they ought to provide [40]. This interpretation may help explain why burnout was more strongly associated with turnover intention at higher empathy levels. It should also be noted that, despite the theoretical relevance of these processes, the present models explain a modest proportion of variance in turnover intention (approximately 16% before and 18% after inclusion of the BAT × JSE interaction) and burnout (approximately 23%). This suggests that both outcomes are shaped by a range of factors beyond empathy, and that empathy should be understood as one contextual influence within a broader constellation of individual, organizational, and situational determinants.

In this broader clinical and organizational context, healthcare systems have become increasingly complex and constrained, particularly in the post–COVID‐19 period. Financial pressures, alongside expanded documentation and procedural requirements aimed at ensuring safety and reducing legal risk, have intensified organizational demands [41]. Although these measures are necessary for institutional sustainability, frontline professionals may experience them as constraints that limit ethically grounded, patient‐centered care [42]. Health care is fundamentally guided by altruistic values, yet organizations must operate within finite resources and structural constraints that are often beyond individual control [43]. Within this tension, delivering ideal care becomes increasingly challenging. Addressing this gap requires not only individual resilience but also a shared, organizational form of empathy that acknowledges both ethical aspirations and systemic realities [44]. Accordingly, empathy should be understood not only as an individual trait but as a relational and institutional capacity that must be actively supported within healthcare systems. Reframing empathy education to reflect system‐level constraints may help professionals sustain empathic engagement without amplifying emotional exhaustion or moral compromise [45, 46]. Consistent with this perspective, the present findings support further investigation of organizational approaches to reducing system‐level MD alongside individual‐focused approaches to burnout and turnover intention.

## 5. Implications for Nursing Management

Taken together with previous research, our findings suggest that nursing management should support empathic practice while attending to burnout and the ethical and organizational conditions of care. When highly empathic nurses experience burnout or ethical difficulties, understanding the care they value and the barriers to providing it may help guide support. The most consistent associations involved system‐level MD, highlighting potential organizational priorities: staffing adequacy and workload management, a supportive ethical climate, nurses’ decision‐making autonomy, and organizational support [2, 44, 45]. Correlations among domains also suggest the importance of considering ethical concerns arising in patient care and team relationships. Practical approaches include interprofessional case discussions and team reflection that enable nurses to share concerns, consider responses, and participate in decisions [45]. Empathy education may also incorporate awareness of MD and ways to seek organizational support [13, 14, 46]. These approaches were not evaluated in the present study; further research is needed to assess their feasibility and effectiveness.

## 6. Limitations

Several limitations should be considered. First, as a cross‐sectional study relying on self‐reported data, the present design restricts our ability to draw causal inferences. In addition, because all variables were assessed using self‐report measures collected at a single time point, common method variance cannot be ruled out, and Harman’s single‐factor test alone does not establish its absence.

Second, although self‐report instruments are standard in psychological research, they are subject to potential response biases, including social desirability effects. In addition, unmeasured contextual variables, such as workplace culture, coping strategies, and organizational support, may have influenced the results. Because participants were recruited through a voluntary online research panel and the principal variables were assessed by self‐report, self‐selection and socially desirable responding may have influenced the observed distribution of empathy scores. Because the direction and magnitude of these potential biases cannot be determined from the present data, the value of 76.66 should be regarded as a sample‐specific statistical boundary rather than as a clinical cutoff for empathy, and its generalizability to other nursing populations requires confirmation.

Third, turnover intention was assessed using a single‐item Likert scale. Although this approach is commonly used for clearly defined constructs and has demonstrated acceptable validity in prior research, it may not fully capture the multidimensional nature of turnover intention. Fourth, the survey did not capture unit‐level clinical settings, such as intensive care or emergency departments; therefore, residual confounding related to high‐intensity work environments cannot be excluded.

Finally, potential interactions between system‐level and individual‐level MD were not explicitly modeled. As a result, more complex pathways or moderation may have gone undetected. Future research should employ longitudinal designs to clarify the temporal relationships among empathy, MD, burnout, and turnover intention.

## 7. Conclusion

This study suggests that empathy has multifaceted associations with burnout and turnover intention among nurses. Specifically, empathy showed a negative direct association with burnout and a negative indirect association with turnover intention through burnout, alongside positive indirect associations through system‐level MD. System‐level MD showed the most consistent domain‐specific pattern across the analyses. The positive association between burnout and turnover intention was stronger at higher empathy levels. These findings underscore the importance of considering empathy alongside burnout and the ethical and organizational conditions of care. The cross‐sectional design does not establish temporal or causal relationships.

## Funding

This work was supported by the Japan Society for the Promotion of Science (JSPS) KAKENHI Grant Number 23K09609.

## Disclosure

The funder had no role in the study design, data collection, data analysis or interpretation, or preparation of the manuscript.

## Conflicts of Interest

The authors declare no conflicts of interest.

## Supporting Information

Additional supporting information can be found online in the Supporting Information section.

## Supporting information


**Supporting Information 1** Supporting Tables S1 and S2 present the interaction tests used to assess homogeneity of regression slopes. Supporting Tables S3 and S4 present the covariate‐adjusted sensitivity analyses for the regression and indirect‐association models, respectively. Supporting Table S5 presents the separate‐domain sensitivity analyses of indirect associations and turnover intention.

**Supporting Information 2** STROBE_Checklist.

## Data Availability

The data that support the findings of this study are not publicly available due to ethical restrictions but are available from the corresponding author upon reasonable request.

## References

[1] World Health Organization , State of the World’s Nursing 2020: Investing in Education, Jobs and Leadership, 2020, https://www.who.int/publications/i/item/9789240003279.

[2] Xue K. , Shang J. , Yang C. , Pan L. , Shi H. , and Zeng Y. , Nurses’ Moral Distress and Ethical Climate: A Systematic Review and Meta-Analysis, Nursing Ethics. (November 2025) 32, no. 7, 1981–1997, 10.1177/09697330251350384. 40550472

[3] Bondjers K. , Glad A. K. , Wøien H. et al., Moral Distress and Protective Work Environment for Healthcare Workers During Public Health Emergencies, BMC Medical Ethics. (October 2024) 25, no. 1, 10.1186/s12910-024-01098-w. PMC11443852 39354454

[4] Beheshtaeen F. , Torabizadeh C. , Khaki S. , Abshorshori N. , and Vizeshfar F. , Moral Distress Among Critical Care Nurses Before and During the COVID-19 Pandemic: A Systematic Review, Nursing Ethics. (June 2024) 31, no. 4, 613–634, 10.1177/09697330231221196. 38116787

[5] Gougjehyaran H. G. , Amanollahzadeh A. , and Danaloo M. R. , Invisible Nursing Care: A Concept Analysis, BMC Nursing. (November 2025) 24, no. 1, 10.1186/s12912-025-04082-w. PMC12649001 41291700

[6] Gustavsson M. E. , Juth N. , Von Schreeb J. , and Arnberg F. K. , Moral Distress, Moral Residue, and Associations With Psychological Distress: A Cross-Sectional Study, European Journal of Psychotraumatology. (December 2025) 16, no. 1, 10.1080/20008066.2025.2512677. PMC12180323 40534481

[7] Hojat M. , DeSantis J. , Shannon S. C. et al., The Jefferson Scale of Empathy: A Nationwide Study of Measurement Properties, Underlying Components, Latent Variable Structure, and National Norms in Medical Students, Advances in Health Sciences Education. (December 2018) 23, no. 5, 899–920, 10.1007/s10459-018-9839-9. PMC6245107 29968006

[8] Simou E. , The Growing Importance of Soft Skills in Medical Education in the AI Era: Balancing Humanistic Care and Artificial Intelligence, Int Med Educ. (November 2025) 4, no. 4, 10.3390/ime4040050.

[9] Sahoo S. P. , Empathy Training in Nursing Education: A Review of Experiential Approaches and Outcomes in the Asian Context, Humanist Stud Soc Res. (November 2025) 1, no. 1, 10.22034/hssr.2025.561974.1026.

[10] Lee H. C. , Chiang Y. C. , Chu T. L. , Lin C. T. , Moser D. K. , and Hsiao Y. C. , Beyond Presentation Skills: Effects of a Health Care Presentation Course on Nursing Students, Journal of Nursing Education. (January 2026) 65, no. 1, 12–19, 10.3928/01484834-20250826-01. 41537791

[11] Zhou H. , Relationship Between Empathy and Burnout as Well as Potential Affecting and Mediating Factors From the Perspective of Clinical Nurses: A Systematic Review, BMC Nursing. (January 2025) 24, no. 1, 10.1186/s12912-025-02701-0. PMC11720541 39794782

[12] Ren Y. , Song H. , Li S. , and Xiao F. , Mediating Effects of Nursing Organizational Climate on the Relationships Between Empathy and Burnout Among Clinical Nurses, Journal of Advanced Nursing. (2020) 76, no. 11, 3048–3058, 10.1111/jan.14525. 32885476

[13] Nagle E. , Šuriņa S. , and Griškēviča I. , Healthcare Workers’ Moral Distress During the COVID-19 Pandemic: A Scoping Review, Social Science. (2023) 12, no. 7, 10.3390/socsci12070371.

[14] Riedel P. L. , Kreh A. , Kulcar V. , Lieber A. , and Juen B. , A Scoping Review of Moral Stressors, Moral Distress and Moral Injury in Healthcare Workers During COVID-19, International Journal of Environmental Research and Public Health. (February 2022) 19, no. 3, 10.3390/ijerph19031666. PMC8835282 35162689

[15] Epstein E. G. , Whitehead P. B. , Prompahakul C. , Thacker L. R. , and Hamric A. B. , Enhancing Understanding of Moral Distress: The Measure of Moral Distress for Health Care Professionals, AJOB Empir Bioeth. (2019) 10, no. 2, 113–124, 10.1080/23294515.2019.1586008. 31002584

[16] Epstein E. G. and Hamric A. B. , Moral Distress, Moral Residue, and the Crescendo Effect, Journal of Clinical Ethics. (2009) 20, no. 4, 330–342, 10.1086/jce200920406. 20120853

[17] Orgambídez A. , Borrego Y. , Alcalde F. J. , and Durán A. , Moral Distress and Emotional Exhaustion in Healthcare Professionals: A Systematic Review and Meta-Analysis, Healthcare. (February 2025) 13, no. 4, 10.3390/healthcare13040393. PMC11855070 39997268

[18] Ernst P. , Linden K. , Roczniewska M. et al., Moral Distress Among Maternity and Neonatal Healthcare Workers During the COVID-19 Pandemic in Sweden: Results From the COPE Staff Longitudinal Cohort Study, Acta Obstetricia et Gynecologica Scandinavica. (2025) 104, no. 7, 1399–1409, 10.1111/aogs.15125. PMC12144571 40247755

[19] Petrişor C. , Breazu C. , Doroftei M. , Mărieş I. , and Popescu C. , Association of Moral Distress With Anxiety, Depression, and an Intention to Leave Among Nurses Working in Intensive Care Units During the COVID-19 Pandemic, Healthcare. (October 2021) 9, no. 10, 10.3390/healthcare9101377. PMC8544563 34683057

[20] Wu S. , Sun Y. , Zhong Z. , Li H. , Ding B. , and Deng Q. , The Effect of Moral Distress on Emergency Nurses’ Job Burnout: The Mediating Roles of Hospital Ethical Climate and Moral Resilience, Frontiers in Public Health. (March 2025) 13, 10.3389/fpubh.2025.1562209. PMC11920164 40109433

[21] Sorina E. , Novacescu D. , Barb A. C. et al., From Burnout to Resilience: Addressing Moral Injury in Nursing Through Organizational Innovation in the Post-Pandemic Era, Healthc Basel Switz. (November 2025) 13, no. 21, 10.3390/healthcare13212822. PMC12610207 41228189

[22] Gamvrouli M. , Karanikola M. , Triantafyllou C. , Paschali A. , and Giannakopoulou M. , Levels of Moral Distress, Secondary Traumatic Stress, General Health, and Empathy Among Nursing Staff in Eight Public Hospitals in Greece: A Cross‐Sectional Study, Worldviews on Evidence-Based Nursing. (December 2025) 22, no. 6, 10.1111/wvn.70093. 41267457

[23] Lamiani G. , Dordoni P. , Vegni E. , and Barajon I. , Caring for Critically Ill Patients: Clinicians’ Empathy Promotes Job Satisfaction and Does Not Predict Moral Distress, Frontiers in Psychology. (January 2020) 10, 10.3389/fpsyg.2019.02902. PMC6960200 31969851

[24] Fukase Y. , Ichikura K. , and Tagaya H. , Symptoms and Risk Factors of Depression and PTSD in the Prolonged COVID-19 Pandemic: A Longitudinal Survey Conducted From 2020 to 2022 in Japan, BMC Psychiatry. (March 2023) 23, no. 1, 10.1186/s12888-023-04670-7. PMC10026201 36941574

[25] Muto K. , Yamamoto I. , Nagasu M. , Tanaka M. , and Wada K. , Ojima T. , Japanese Citizens’ Behavioral Changes and Preparedness Against COVID-19: An Online Survey During the Early Phase of the Pandemic, PLoS One. (June 2020) 15, no. 6, 10.1371/journal.pone.0234292. PMC7289361 32525881

[26] Kataoka H. U. , Koide N. , Ochi K. , Hojat M. , and Gonnella J. S. , Measurement of Empathy Among Japanese Medical Students: Psychometrics and Score Differences by Gender and Level of Medical Education, Academic Medicine. (September 2009) 84, no. 9, 1192–1197, 10.1097/ACM.0b013e3181b180d4. 19707056

[27] Otsuka T. , Sakaguchi K. , Houchens N. et al., Empathy Among Physicians and Nurses in Japan: A Nationwide Cross-Sectional Study, Journal of General Internal Medicine. (May 2024) 39, no. 6, 960–968, 10.1007/s11606-024-08620-1. PMC11074055 38277022

[28] Fujii T. , Katayama S. , Miyazaki K. et al., Translation and Validation of the Japanese Version of the Measure of Moral Distress for Healthcare Professionals, Health and Quality of Life Outcomes. (April 2021) 19, 10.1186/s12955-021-01765-1. PMC8045393 33849571

[29] Sakakibara K. , Shimazu A. , Toyama H. , and Schaufeli W. B. , Validation of the Japanese Version of the Burnout Assessment Tool, Frontiers in Psychology. (August 2020) 11, 10.3389/fpsyg.2020.01819. PMC7431961 32849072

[30] Hayes A. F. , Introduction to Mediation, Moderation, and Conditional Process Analysis: A Regression-based Approach, 2022, 3rd edition, Guilford Press, New York, NY, https://ci.nii.ac.jp/ncid/BD04210088.

[31] He S. , Sultana R. , Anantham D. et al., Empathy Levels Among Healthcare Professionals: An Asian Multi-Professional Cross-Sectional Study, Cureus. (2024) 16, no. 2, 10.7759/cureus.53750. PMC10921128 38465054

[32] Kuo J. , Cheng J. , Chen Y. , Livneh H. , and Tsai T. , An Exploration of Empathy and Correlates Among Taiwanese Nurses, Jpn J Nurs Sci. (2012) 9, no. 2, 169–76, 10.1111/j.1742-7924.2011.00199.x. 23181885

[33] Ghaedi F. , Ashouri E. , Soheili M. , and Sahragerd M. , Nurses’ Empathy in Different Wards: A Cross-Sectional Study, Iran J Nurs Midwifery Res. (2020) 25, no. 2, 117–121. 10.4103/ijnmr.IJNMR_84_19 PMC7055183 32195156

[34] Lin H. , Probing Two-Way Moderation Effects: A Review of Software to Easily Plot Johnson-Neyman Figures, Struct Equ Model Multidiscip J. (2020) 27, no. 3, 494–502, 10.1080/10705511.2020.1732826.

[35] Duarte J. and Pinto-Gouveia J. , Empathy and Feelings of Guilt Experienced by Nurses: A Cross-Sectional Study of Their Role in Burnout and Compassion Fatigue Symptoms, Appl Nurs Res. (2017) 35, 42–7, 10.1016/j.apnr.2017.02.006. 28532725

[36] Farmahini Farahani M. , Jaberi K. , and Purfarzad Z. , Ramos-Pichardo J. D. , Workplace Spirituality, Compassion Satisfaction, Burnout, and Secondary Traumatic Stress: A Cross‐Sectional Study in Iranian Nurses, Perspectives in Psychiatric Care. (2023) 2023, no. 1, 10.1155/2023/7685791.

[37] Ni W. , Xia M. , Jing M. , Zhu S. , and Li L. , The Relationship Between Professional Quality of Life and Work Environment Among ICU Nurses in Chinese: A Cross-Sectional Study, Frontiers in Public Health 11. (2023) 10.3389/fpubh.2023.1104853. PMC10192618 37213646

[38] Pepito J. A. , Babate F. J. , Soriano G. P. et al., Digital Empathy in Nursing: Developing a Situated Model for Compassionate Care in Technology‐Mediated Environments, Nurs Inq. (April 2026) 33, no. 2, 10.1111/nin.70102. 41950368

[39] Chen A. M. H. , Blakely M. L. , Daugherty K. K. , Kiersma M. E. , Meny L. M. , and Pereira R. , Meaningful Connections: Exploring the Relationship Between Empathy and Professional Identity Formation, American Journal of Pharmaceutical Education. (2024) 88, no. 8, 10.1016/j.ajpe.2024.100725. 38810953

[40] Maslach C. and Leiter M. P. , Understanding the Burnout Experience: Recent Research and Its Implications for Psychiatry, World Psychiatry. (2016) 15, no. 2, 103–11, 10.1002/wps.20311. PMC4911781 27265691

[41] Friganovic A. , Boskovic S. , Krupa Nurcek S. , Kovacevic I. , Kosydar-Bochenek J. , and Filipovic B. , Editorial: Patient and Medical Staff Safety and Healthy Work Environment in the 21st Century, Front Public Health. (2025) 13, 10.3389/fpubh.2025.1677117. PMC12539444 41127605

[42] Narayan M. C. and Mallinson R. K. , Home Health Nurses’ Journey Toward Culture-Sensitive/Patient-Centered Skills: A Grounded Theory Study, Home Health Care Manag Pract. (2022) 34, no. 1, 24–34, 10.1177/10848223211027860.

[43] Morley G. , Ives J. , Bradbury-Jones C. , and Irvine F. , What is ‘Moral Distress’? A Narrative Synthesis of the Literature, Nurs Ethics. (2019) 26, no. 3, 646–62, 10.1177/0969733017724354. PMC6506903 28990446

[44] Dzau V. J. , Kirch D. , and Nasca T. , Preventing a Parallel Pandemic—A National Strategy to Protect Clinicians’ Well-Being, N Engl J Med. (2020) 383, no. 6, 513–515, 10.1056/NEJMp2011027. 32402153

[45] Nielsen C. L. , Lindhardt C. L. , Näslund‐Koch L. , Frandsen T. F. , Clemensen J. , and Timmermann C. , What is the State of Organisational Compassion‐Based Interventions Targeting to Improve Health Professionals’ Well‐Being? Results of a Systematic Review, J Adv Nurs. (2025) 81, no. 5, 2246–76, 10.1111/jan.16484. PMC11967289 39373033

[46] Robinson R. , Meluski K. , Hellem T. et al., Rapid Scoping Review: Empathy in Health Sciences Curriculum, Healthcare. (2023) 11, no. 10, 10.3390/healthcare11101429. PMC10218519 37239715

