# Supplementary material for: Empathy as a Conditional Resource: System‐Level Moral Distress as an Indirect Pathway in the Associations of Empathy with Burnout and Turnover Intention
Source: J Nurs Manag. 2026 Oct 21;2026:9665722. doi: 10.1155/jonm/9665722 (PMC13687653; doi:10.1155/jonm/9665722)
Supplement: Supplementary file 1 [file JONM-2026-9665722-s001.docx]

**STROBE Checklist for Cross-Sectional Studies — Completed**

**Journal of Nursing Management — Manuscript ID 9665722**

*“Empathy as a Conditional Resource: System-Level Moral Distress as an Indirect Pathway in the Associations of Empathy With Burnout and Turnover Intention”*

Page and line references correspond to the revised clean manuscript prepared for resubmission on August 22, 2026.

| **Section** | **Item** | **STROBE reporting element** | **Location in revised manuscript** | **Status** |
| --- | --- | --- | --- | --- |
| Title and abstract | 1a | Study design identified using a standard design term. | p. 2, line 45 (cross-sectional web-based survey). | Reported |
| Title and abstract | 1b | Abstract provides a balanced summary of methods and findings. | p. 2, lines 35–66. | Reported |
| Introduction | 2 | Scientific background and rationale are explained. | pp. 3–4, lines 69–121. | Reported |
| Introduction | 3 | Specific study objective is stated. | p. 4, lines 122–124. | Reported |
| Methods | 4 | Key elements of the study design are presented early. | p. 4, lines 127–130; design also stated in Abstract, p. 2, line 45. | Reported |
| Methods | 5 | Setting, location, and relevant dates/data-collection period are described. | pp. 4–6, lines 127–185 (Japan; July 2025; nationwide online panel and field procedures). | Reported |
| Methods | 6a | Eligibility criteria and participant source/selection methods are described. | pp. 5–6, lines 139–185 (sampling frame, eligibility criteria, and participant selection procedures). | Reported |
| Methods | 7 | Outcomes, predictors, potential confounders, and effect modifiers are defined. | pp. 6–8, lines 188–225 and 268–281; demographic/occupational covariates are listed at lines 188–191 and modeled at lines 268–281. | Reported |
| Methods | 8 | Data sources and measurement methods are described for variables of interest. | pp. 6–7, lines 194–225 (JSE, MMD-HP, BAT, turnover intention). | Reported |
| Methods | 9 | Efforts to address potential bias are described. | pp. 5–8, lines 146–185, 236–240, and 268–281; limitations and possible selection/response bias discussed at p. 14, lines 475–492. | Reported |
| Methods | 10 | Study-size determination is explained. | p. 4, lines 131–138. | Reported |
| Methods | 11 | Handling/grouping of quantitative variables is described. | pp. 6–8, lines 194–225 and 274–278; nursing experience was entered as ≥15 vs <15 years in the complete-case sensitivity model; category boundaries also appear in Table 1 and Supplementary Table S3. | Reported |
| Methods | 12a | Statistical methods, including methods used to control for confounding, are described. | pp. 7–9, lines 227–282. | Reported |
| Methods | 12b | Methods for subgroup/interaction analyses are described. | p. 8, lines 253–267; Supplementary Tables S1–S2. | Reported |
| Methods | 12c | Handling of missing data is explained. | p. 8, lines 274–281 (45 participants missing nursing experience and managerial position; complete-case Model 4, n = 367). | Reported |
| Methods | 12d | Analytical handling of the sampling strategy is described, if applicable. | p. 5, lines 159–165 (non-probability online-panel sample; no stratification or quota sampling). No survey-weighted analysis was applied. | N/A / described |
| Methods | 12e | Sensitivity analyses are described. | pp. 8–9, lines 268–282; Supplementary Tables S3–S4. | Reported |
| Results | 13a | Numbers of individuals at key study stages are reported. | pp. 5–6, lines 159–185 (approximately 30,000 selected for invitation; 424 eligible/complete after initial QC; 12 rapid responses excluded; 412 analyzed). | Reported |
| Results | 13b | Reasons for non-participation/exclusion are provided where available. | pp. 5–6, lines 149–155 and 173–185 (eligibility termination and data-quality exclusions, including 12 rapid responses, are described). | Reported as available |
| Results | 13c | Use of a participant flow diagram is considered. | No flow diagram was used; recruitment and exclusions are described narratively at pp. 5–6, lines 159–185. | Not used |
| Results | 14a | Participant characteristics and relevant covariates are reported. | p. 9, lines 291–297; Tables 1–2. | Reported |
| Results | 14b | Numbers with missing data are reported. | p. 8, lines 274–278; Table 1 reports 45 missing values for working position and nursing experience due to routing discrepancy. | Reported |
| Results | 15 | Outcome summary data are reported. | p. 9, lines 292–297; Table 2 (turnover intention M = 4.28, SD = 1.81). | Reported |
| Results | 16a | Main unadjusted and adjusted estimates, with precision where applicable, are reported and adjusted covariates are identified. | pp. 9–10, lines 305–349; Tables 4–6; adjusted sensitivity results in Supplementary Tables S3–S4. | Reported |
| Results | 16b | Category boundaries are given when continuous variables are categorized. | p. 8, lines 274–278; Table 1 and Supplementary Table S3 (nursing experience ≥15 vs <15 years; hospital-size categories). | Reported |
| Results | 16c | Absolute-risk translation is considered when relevant. | Not applicable: analyses estimate regression/indirect associations rather than relative risks over time. | N/A |
| Results | 17 | Other analyses, including interaction and sensitivity analyses, are reported. | pp. 9–10, lines 313–349; Supplementary Tables S1–S4; Figures 2–3. | Reported |
| Discussion | 18 | Key results are summarized in relation to study objectives. | p. 11, lines 353–366. | Reported |
| Discussion | 19 | Study limitations and potential sources of bias/imprecision are discussed. | pp. 14–15, lines 475–501. | Reported |
| Discussion | 20 | Overall interpretation is cautious and considers objectives, limitations, multiplicity, and prior evidence. | pp. 11–14, lines 353–492; caution regarding causal/threshold interpretation at p. 13, lines 429–438 and p. 14, lines 484–492. | Reported |
| Discussion | 21 | Generalisability/external validity is discussed. | p. 11, lines 367–373; p. 14, lines 487–492. | Reported |
| Other information | 22 | Funding source and funder role are reported. | p. 15, lines 520–522 (funding source and statement that the funder had no role in study design, data collection, data analysis or interpretation, or manuscript preparation). | Reported |

**Note.** Prepared using the STROBE Statement checklist for cross-sectional studies (version 4). Item descriptions are concise paraphrases for submission tracking.
