# Supplementary material for: Empathy as a Conditional Resource: System‐Level Moral Distress as an Indirect Pathway in the Associations of Empathy with Burnout and Turnover Intention
Source: J Nurs Manag. 2026 Oct 21;2026:9665722. doi: 10.1155/jonm/9665722 (PMC13687653; doi:10.1155/jonm/9665722)
Supplement: Supplementary file 2 [file JONM-2026-9665722-s002.docx]

**Supplementary Table S1. Tests of the Homogeneity of Regression Slopes: Interactions Between Empathy and Moral-Distress Subdomains With Burnout as the Dependent Variable**

| **Interaction** | **F (HC3)** | **p** |
| --- | --- | --- |
| Clinical/patient-level MMD-HP × JSE | 0.0874 | 0.768 |
| System-level MMD-HP × JSE | 0.0094 | 0.923 |
| Team/personal threat/vulnerability MMD-HP × JSE | 0.3117 | 0.577 |
| Team/patient interaction MMD-HP × JSE | 0.3592 | 0.549 |

*Note.* JSE = Jefferson Scale of Empathy; MMD-HP = Measure of Moral Distress for Healthcare Professionals.

**Supplementary Table S2. Tests of the Homogeneity of Regression Slopes: Interactions Between Empathy and Candidate Mediators With Turnover Intention as the Dependent Variable**

| **Interaction** | **F (HC3)** | **p** |
| --- | --- | --- |
| Clinical/patient-level MMD-HP × JSE | 0.1959 | 0.658 |
| System-level MMD-HP × JSE | 0.0594 | 0.807 |
| Team/personal threat/vulnerability MMD-HP × JSE | 1.1972 | 0.274 |
| Team/patient interaction MMD-HP × JSE | 0.0348 | 0.852 |
| BAT × JSE | 7.1036 | 0.008 |

*Note.* BAT = Burnout Assessment Tool; JSE = Jefferson Scale of Empathy; MMD-HP = Measure of Moral Distress for Healthcare Professionals.

**Supplementary Table S3. Sensitivity Analyses: Hierarchical Multiple Regression Models With Turnover Intention as the Dependent Variable, Including Demographic and Occupational Covariates**

| **Predictor** | **Model 1** | **Model 2** | **Model 3** | **Model 4** |
| --- | --- | --- | --- | --- |
| Constant | 1.252 | 1.395 | 1.326 | 1.773 |
| Empathy (JSE) | 0.002 | 0.003 | 0.003 | 0.003 |
| Clinical/patient-level MD | 0.004 | 0.003 | 0.004 | 0.002 |
| System-level MD | 0.019* | 0.020** | 0.019* | 0.021* |
| Team/personal threat (TPV-MD) | −0.011 | −0.013 | −0.012 | −0.011 |
| Team/patient interaction (TPI-MD) | −0.004 | −0.002 | −0.003 | −0.002 |
| Burnout (BAT) | 0.033*** | 0.032*** | 0.032*** | 0.030*** |
| BAT × JSE | 0.001** | 0.001** | 0.001** | 0.001** |
| Age | 0.003 | 0.008 | 0.009 | 0.005 |
| Sex (female) | 0.213 | 0.123 | 0.129 | 0.008 |
| Married |  | −0.442* | −0.435* | −0.401 |
| Has child(ren) |  | −0.139 | −0.144 | −0.240 |
| Hospital beds: 100–499 |  |  | −0.234 | −0.352 |
| Hospital beds: ≥500 |  |  | −0.207 | −0.299 |
| Night shift |  |  | 0.072 | 0.024 |
| Inpatient setting |  |  | 0.121 | 0.167 |
| Nursing experience ≥15 years |  |  |  | 0.281 |
| Managerial position |  |  |  | −0.345 |
| N | 412 | 412 | 412 | 367 |
| R² | .179 | .199 | .202 | .214 |
| Adjusted R² | .160 | .177 | .172 | .175 |
| ΔR² | .004 | .020 | .004 | — |
| Johnson–Neyman boundary (JSE) | 76.91 | 78.27 | 78.40 | 80.66 |

*Note.* Entries are unstandardized coefficients (b); inference is based on HC3 standard errors. BAT and JSE were entered in their original scale units; BAT and JSE centered at the full-sample means were used only to compute the BAT × JSE product term. The unadjusted primary interaction model is reported in the main manuscript and is not repeated here. Model 1 adjusted for age and sex; Model 2 additionally adjusted for marital and parental status; Model 3 additionally adjusted for hospital size, night-shift work, and inpatient setting; and Model 4 additionally included managerial position and nursing experience ≥15 years in the complete-case sample. Models 1–3 used N = 412; Model 4 used n = 367 because managerial position and nursing experience were unavailable for the same 45 participants owing to a screening-system routing discrepancy. All variance inflation factors were below 5. Johnson–Neyman boundaries were calculated from the fitted BAT and BAT × JSE coefficients and their HC3 covariance matrix using t inference and the covariate set of the corresponding model. * p < .05. ** p < .01. *** p < .001.

**Supplementary Table S4. Sensitivity Analyses of Indirect Pathways Linking Empathy With Turnover Intention**

| **Pathway** | **JSE** | **Model 1 + Age, sex N = 412** | **Model 2 + Marital, parental N = 412** | **Model 3 Full-sample adjusted N = 412** | **Model 4 Complete-case adjusted n = 367** |
| --- | --- | --- | --- | --- | --- |
| ***Panel A. Specific indirect associations through the four MMD-HP subdomains*** | | | | | |
| JSE → clinical/patient-level MD → TI | – | 0.000736 (0.001769) [−0.002627, 0.004638] | 0.000581 (0.001822) [−0.003308, 0.004384] | 0.000605 (0.001563) [−0.002534, 0.003885] | 0.000374 (0.002009) [−0.003556, 0.004616] |
| JSE → system-level MD → TI | – | 0.005634 (0.002921) [0.000834, 0.012046] | 0.005771 (0.002909) [0.000945, 0.012283] | 0.004785 (0.002583) [0.000654, 0.010566] | 0.005386 (0.002996) [0.000661, 0.012262] |
| JSE → team/personal threat MD → TI | – | 0.000377 (0.001140) [−0.001991, 0.002816] | 0.000384 (0.001190) [−0.002211, 0.002884] | 0.000629 (0.001130) [−0.001725, 0.003087] | 0.000403 (0.001158) [−0.002199, 0.002826] |
| JSE → team/patient interaction MD → TI | – | −0.000100 (0.000724) [−0.001840, 0.001243] | −0.000052 (0.000682) [−0.001723, 0.001260] | −0.000012 (0.000624) [−0.001461, 0.001223] | −0.000071 (0.000832) [−0.002128, 0.001486] |
| ***Panel B. Conditional indirect association through burnout*** | | | | | |
| JSE → BAT → TI | 79 | −0.005479 (0.002497) [−0.010773, −0.000809] | −0.004975 (0.002512) [−0.010388, −0.000341] | −0.005037 (0.002504) [−0.010442, −0.000447] | −0.004223 (0.002602) [−0.009836, 0.000551] |
|  | 86 | −0.007454 (0.002400) [−0.012671, −0.003240] | −0.007077 (0.002404) [−0.012363, −0.002948] | −0.007195 (0.002391) [−0.012425, −0.003069] | −0.006305 (0.002429) [−0.011626, −0.002176] |
|  | 108 | −0.013661 (0.003905) [−0.022474, −0.007384] | −0.013686 (0.003916) [−0.022755, −0.007388] | −0.013975 (0.003922) [−0.022631, −0.007543] | −0.012846 (0.003946) [−0.021915, −0.006437] |
| ***Panel C.*** ***Conditional serial indirect associations through each MMD-HP domain and burnout in the parallel mediation framework*** | | | | | |
| JSE → clinical/patient-level MD → BAT → TI | 79 | −0.000116 (0.000327) [−0.000895, 0.000485] | −0.000112 (0.000310) [−0.000821, 0.000456] | −0.000109 (0.000267) [−0.000732, 0.000377] | −0.000175 (0.000356) [−0.001078, 0.000402] |
|  | 86 | −0.000158 (0.000419) [−0.001093, 0.000636] | −0.000159 (0.000409) [−0.001053, 0.000608] | −0.000156 (0.000349) [−0.000944, 0.000515] | −0.000261 (0.000461) [−0.001348, 0.000589] |
|  | 108 | −0.000289 (0.000745) [−0.001879, 0.001211] | −0.000308 (0.000762) [−0.001954, 0.001192] | −0.000302 (0.000643) [−0.001697, 0.001032] | −0.000532 (0.000863) [−0.002385, 0.001186] |
| JSE → system-level MD → BAT → TI | 79 | 0.001362 (0.000790) [0.000158, 0.003223] | 0.001248 (0.000772) [0.000062, 0.002980] | 0.000976 (0.000649) [0.000044, 0.002466] | 0.000907 (0.000716) [−0.000094, 0.002640] |
|  | 86 | 0.001853 (0.000845) [0.000543, 0.003834] | 0.001775 (0.000835) [0.000462, 0.003651] | 0.001394 (0.000722) [0.000292, 0.003039] | 0.001353 (0.000776) [0.000233, 0.003207] |
|  | 108 | 0.003396 (0.001372) [0.001168, 0.006455] | 0.003433 (0.001407) [0.001181, 0.006624] | 0.002708 (0.001223) [0.000728, 0.005437] | 0.002757 (0.001313) [0.000660, 0.005834] |
| JSE → team/personal threat MD → BAT → TI | 79 | −0.000130 (0.000377) [−0.000885, 0.000692] | −0.000105 (0.000341) [−0.000799, 0.000641] | −0.000192 (0.000354) [−0.000954, 0.000537] | −0.000121 (0.000379) [−0.000921, 0.000708] |
|  | 86 | −0.000177 (0.000482) [−0.001169, 0.000853] | −0.000150 (0.000449) [−0.001055, 0.000799] | −0.000274 (0.000470) [−0.001271, 0.000693] | −0.000181 (0.000498) [−0.001215, 0.000892] |
|  | 108 | −0.000324 (0.000855) [−0.002123, 0.001387] | −0.000290 (0.000832) [−0.002025, 0.001383] | −0.000533 (0.000895) [−0.002463, 0.001167] | −0.000369 (0.000945) [−0.002399, 0.001552] |
| JSE → team/patient interaction MD → BAT → TI | 79 | −0.000069 (0.000206) [−0.000612, 0.000258] | −0.000062 (0.000190) [−0.000552, 0.000210] | −0.000011 (0.000171) [−0.000448, 0.000292] | −0.000094 (0.000223) [−0.000687, 0.000180] |
|  | 86 | −0.000093 (0.000262) [−0.000746, 0.000339] | −0.000088 (0.000245) [−0.000712, 0.000302] | −0.000015 (0.000226) [−0.000564, 0.000405] | −0.000140 (0.000286) [−0.000860, 0.000268] |
|  | 108 | −0.000171 (0.000459) [−0.001250, 0.000663] | −0.000170 (0.000442) [−0.001254, 0.000621] | −0.000030 (0.000422) [−0.000999, 0.000829] | −0.000285 (0.000520) [−0.001539, 0.000582] |

*Note. Entries are unstandardized indirect-association estimates presented as estimate (bootstrap SE) [95% percentile bootstrap CI], based on 5,000 nonparametric case-resampling bootstrap samples. Statistical significance was determined using unrounded confidence limits. The corresponding unadjusted primary estimates are reported in the main tables and are not repeated here. Model numbering corresponds to Supplementary Table S3. Panel A reports specific indirect associations from JSE to TI through each MMD-HP subdomain using the same equation specification as Panels B and C. BAT and BAT × JSE were included only in the TI equation, not in the first-stage MMD-HP equations. Panel B reports the conditional indirect association through burnout, with MMD-HP subdomains included as covariates. Panel C reports domain-specific conditional serial indirect associations through each MMD-HP domain and burnout in parallel mediation framework; each MMD-HP equation included JSE and the designated background covariates without the other MMD-HP domains, while the BAT and TI equations included all four MMD-HP domains and the BAT × JSE term was included only in the TI equation. JSE values 79, 86, and 108 correspond to the 16th, 50th, and 84th percentiles in the complete-case sample (n = 367) and were held fixed across analyses. JSE = Jefferson Scale of Empathy; MMD-HP = Measure of Moral Distress for Healthcare Professionals; BAT = Burnout Assessment Tool; TI = turnover intention; CI = confidence interval. Six decimal places are retained to make small differences across models and confidence limits near zero visible.*

**Supplementary Table S5. Separate-Domain Sensitivity Analyses of Indirect Associations and Turnover Intention**

***Panel A. Separate-domain regression models for turnover intention***

| MMD-HP domain | B | HC3 SE | p | Bonferroni-adjusted p |
| --- | --- | --- | --- | --- |
| Clinical/patient-level MD | .0124 | .00545 | .0238 | .0951 |
| System-level MD | .0132 | .00440 | .0029 | .0117 |
| Team/personal threat MD | .0056 | .00544 | .3027 | 1.0000 |
| Team/patient interaction MD | .0117 | .00746 | .1160 | .4640 |

***Panel B. Domain-specific indirect associations***

| **MMD-HP domain** | **JSE → MMD → BAT** | | **JSE → MMD → TI** | |
| --- | --- | --- | --- | --- |
| Clinical/patient-level MD | 0.0539 (0.0228) 95% [0.0131, 0.1036] 98.75% [0.0041, 0.1200] | | 0.0024 (0.0014) 95% [0.0002, 0.0054] 98.75% [−0.0003, 0.0065] | |
| System-level MD | 0.0774 (0.0257) 95% [0.0314, 0.1317] 98.75% [0.0194, 0.1482] | | 0.0039 (0.0017) 95% [0.0010, 0.0077] 98.75% [0.0004, 0.0089] | |
| Team/personal threat MD | −0.0118 (0.0247) 95% [−0.0600, 0.0391] 98.75% [−0.0752, 0.0513] | | −0.0002 (0.0007) 95% [−0.0020, 0.0008] 98.75% [−0.0030, 0.0014] | |
| Team/patient interaction MD | 0.0072 (0.0188) 95% [−0.0286, 0.0472] 98.75% [−0.0388, 0.0595] | | 0.0002 (0.0007) 95% [−0.0013, 0.0017] 98.75% [−0.0021, 0.0023] | |
| *Panel C* ***Separate-domain conditional serial indirect associations: JSE → MMD → BAT → TI*** | | | | |
| **MMD-HP domain** | **JSE = 79** | **JSE = 86** | | **JSE = 108** |
| Clinical/patient-level MD | 0.001201 (0.000724) 95% [0.000147, 0.002918] 98.75% [0.000007, 0.003574] | 0.001637 (0.000808) 95% [0.000344, 0.003487] 98.75% [0.000109, 0.004198] | | 0.003007 (0.001349) 95% [0.000720, 0.005945] 98.75% [0.000235, 0.007229] |
| System-level MD | 0.001533 (0.000875) 95% [0.000199, 0.003558] 98.75% [−0.000024, 0.004270] | 0.002111 (0.000904) 95% [0.000651, 0.004206] 98.75% [0.000385, 0.004946] | | 0.003925 (0.001402) 95% [0.001623, 0.007019] 98.75% [0.001040, 0.008333] |
| Team/personal threat MD | −0.000283 (0.000618) 95% [−0.001538, 0.001001] 98.75% [−0.001962, 0.001645] | −0.000383 (0.000822) 95% [−0.002042, 0.001267] 98.75% [−0.002597, 0.001862] | | −0.000698 (0.001538) 95% [−0.003969, 0.002128] 98.75% [−0.005238, 0.002991] |
| Team/patient interaction MD | 0.000167 (0.000467) 95% [−0.000640, 0.001224] 98.75% [−0.000956, 0.001736] | 0.000230 (0.000615) 95% [−0.000913, 0.001532] 98.75% [−0.001302, 0.002071] | | 0.000426 (0.001131) 95% [−0.001790, 0.002715] 98.75% [−0.002705, 0.003493] |
| *Note. Each MMD-HP domain was examined separately (N = 412), without simultaneous adjustment for the other MMD-HP domains. Panel A reports four separate turnover-intention regression models, each including one MMD-HP domain, JSE, BAT, and the BAT × JSE interaction, with HC3 heteroskedasticity-consistent standard errors and t tests using residual degrees of freedom; Bonferroni-adjusted p values were calculated from unrounded p values for four focal domain tests (α = .05/4 = .0125). Panel B reports domain-specific indirect associations. For the JSE → MMD → BAT associations, the focal MMD-HP equation included JSE, and the BAT equation included JSE and the focal MMD-HP domain. For the JSE → MMD → TI associations, the focal MMD-HP equation included JSE, whereas the TI equation included JSE, the focal MMD-HP domain, BAT, and the BAT × JSE interaction term. Panel C reports the corresponding separate-domain conditional serial indirect associations; the focal MMD-HP equation included JSE, the BAT equation included JSE and the focal MMD-HP domain, and the TI equation included JSE, the focal MMD-HP domain, BAT, and the BAT × JSE interaction term. Entries in Panels B and C are unstandardized estimates (bootstrap SE), followed by conventional 95% and Bonferroni-adjusted 98.75% percentile bootstrap CIs based on 5,000 nonparametric case-resampling bootstrap samples. JSE values of 79, 86, and 108 correspond to the 16th, 50th, and 84th percentiles in the complete-case sample (n = 367), respectively, and were held fixed across analyses. BAT = Burnout Assessment Tool; JSE = Jefferson Scale of Empathy; MMD-HP = Measure of Moral Distress for Healthcare Professionals; TI = turnover intention; CI = confidence interval.* | | | | |
